# A study on the distribution of 37 well conserved families of C2H2 zinc finger genes in eukaryotes

**DOI:** 10.1186/1471-2164-14-420

**Published:** 2013-06-24

**Authors:** Arun Seetharam, Gary W Stuart

**Affiliations:** 1 Department of Biology, Indiana State University, Terre Haute, IN, 47809, USA; 2 Bioinformatics Core, Purdue University, West Lafayette, IN, 47906, USA

**Keywords:** C2H2 Zinc Finger Genes, Family Expansion, Orthologs Detection, HMM, RBH

## Abstract

**Background:**

The C2H2 zinc-finger (ZNF) containing gene family is one of the largest and most complex gene families in metazoan genomes. These genes are known to exist in almost all eukaryotes, and they constitute a major subset of eukaryotic transcription factors. The genes of this family usually occur as clusters in genomes and are thought to have undergone a massive expansion in vertebrates by multiple tandem duplication events (BMC Evol Biol 8:176, 2008).

**Results:**

In this study, we combined two popular approaches for homolog detection, Reciprocal Best Hit (RBH) (Proc Natl Acad Sci USA 95:6239–6244, 1998) and Hidden–Markov model (HMM) profiles search (Bioinformatics 14:755-763, 1998), on a diverse set of complete genomes of 124 eukaryotic species ranging from excavates to humans to identify all detectable members of 37 C2H2 ZNF gene families. We succeeded in identifying 3,890 genes as distinct members of 37 C2H2 gene families. These 37 families are distributed among the eukaryotes as progressive additions of gene blocks with increasing complexity of the organisms. The first block featuring the protists had 7 families, the second block featuring plants had 2 families, the third block featuring the fungi had 2 families (one of which was also present in plants) and the final block consisted of metazoans with 25 families. Among the metazoans, the simpler unicellular metazoans had just 15 of the 25 families while most of the bilaterians had all 25 families making up a total of 37 families. Multiple potential examples of lineage-specific gene duplications and gene losses were also observed.

**Conclusions:**

Our hybrid approach combines features of the both RBH and HMM methods for homolog detection. This largely automated technique is much faster than manual methods and is able to detect homologs accurately and efficiently among a diverse set of organisms. Our analysis of the 37 evolutionarily conserved C2H2 ZNF gene families revealed a stepwise appearance of ZNF families, agreeing well with the phylogenetic relationship of the organisms compared and their presumed stepwise increase in complexity (Science 300:1694, 2003).

## Background

The morphological complexity of organisms can be, to a certain extent, assigned to the transcription factors that control expression of various genes such as those that control signal transduction, cell growth, differentiation, and development [1]. One such family of transcription factors is the Zinc Finger (ZNF) protein family, which is the largest family of DNA-binding transcription factors in eukaryotes. Of these ZNF proteins, the C2H2 type of zinc finger proteins remains the largest group [2]. This group is characterized by zinc finger domains, consisting of 20-30 amino acid residues with a zinc ion coordinated by 2 cysteine and 2 histidine residues. C2H2 ZNF proteins often contain more than one such finger as tandem repeats. These proteins are known to exist in prokaryotes and eukaryotes and are most common in mammals. It is estimated that more than 700 C2H2 ZNF genes exist in humans accounting for more than 2 per cent of the total human genes [3]. Most of these C2H2 ZNF proteins act by binding DNA duplexes using their zinc finger motifs and are involved in controlling expression of their target genes. Some C2H2 ZNF proteins also play roles as either subunits of transcription proteins, splicing factors, or DNA damage repair proteins [4]. It is reasonable to assume that as morphologically simpler organisms evolved increasing numbers of genes, they must also have developed new control genes, including additional ZNF genes, to evolve into more complex organisms.

With the advent of “next generation” sequencing methods and the explosive growth of sequence databases, faster and more reliable methods for identification of gene family members, including the C2H2 ZNF genes, are of great interest. The study of the evolution of the C2H2 ZNF genes in various genomes may help to elucidate their possible role in the functions associated with speciation. Homolog prediction is one of the most vital steps in the functional annotation of genomes. The correct identification of homologs and putative orthologs greatly facilitates the accuracy of downstream analysis such as phylogenetic tree construction, protein structure prediction, prediction of protein-protein interaction, and species classification [5]. An effective and commonly used method of homolog/ortholog prediction is Reciprocal-best-BLAST-hits (RBH) [6,7], where genes from two species are homologs and potential orthologs if they are both best BLAST hits when the gene from one genome is used to search the other genome. Although RBH is an effective procedure, potential homologs in multi-member families might be missed due to the restricted amount of information about the gene family in question that is present in just two sequences. More sophisticated methods based on Hidden–Markov models (HMM) [8] can also be applied and are easily automated for homolog detection [5,9]. In the HMM method, each family is typically described by one or more information-rich HMM profiles that can be used to efficiently scan entire genomes for matches. This approach in general is very sensitive in detecting homologs and can be applied for large-scale, genome-level detection [5]. Homolog prediction is especially difficult when multiple related gene families are considered, as exemplified by the many diverse C2H2 ZNF gene families [2]. The high baseline of similarity among the families and subfamilies of C2H2 ZNF genes, along with their large numbers makes automated detection and assignment of C2H2 ZNF genes a challenge [2]. Many previous studies have successfully used these methods to uncover a large number of C2H2 ZNF gene families. The most prominent of these provide a comprehensive cataloging of human KRAB-associated ZNF genes that were conserved in mouse, dog and chimpanzee [10-12], a description of the SysZNF database for all the C2H2 ZNF genes of human and mouse [13] and a study on Zinc Finger Associated Domain (ZAD) type C2H2 ZNF gene families in arthropods [14]. All these methods either used HMM profiles generated from the C2H2 ZNF motif or the pfam domain (PF00096) to scan proteomes and identify putative C2H2 ZNF genes as the first step. Identified genes were then validated using BLAST or other related methods. In our approach for gene homolog detection, we combined both RBH and HMM methods in a similar way. But instead of using the C2H2 domain for scanning, we used HMM profiles generated from the C2H2 ZNF gene families for the initial scanning step. The method is analogous to the existing method of orthology detection in expressed sequence tags (EST) called HaMStR (Hidden Markov Model based Search for Orthologs using Reciprocity) [15]. Like our method HaMStR also uses the forward Hidden Markov Model and reverse BLAST search to extend existing ortholog clusters with sequences from additional taxa. However, unlike HaMStR, which used a large number of core orthologs as the reference set, our method only used a targeted set of ortholog families that were manually identified from 4 different species proteomes.

To understand the complex evolution of these C2H2 ZNF gene families, we undertook a survey to identify the different members of C2H2 ZNF family genes from all the eukaryotes that represent different taxa in the Tree of Life. We used the previously identified C2H2 ZNF genes of bilaterians (*Daphnia*, human, worm and fly) as our starting point for analyses [4]. These families were originally identified as conserved C2H2 zinc-finger gene families in bilaterians and were classified based on sequence identity at defined sites [2]. We used the conserved gene families of bilaterians to scan other domains of the Tree of Life because we assumed that shared ancestry of these families in bilaterians could be extended to the lower eukaryotic domain, and that this might serve to identify the approximate point of origin of these gene families within the phylogeny. Also, the availability of well annotated genomes for bilaterians provided high confidence in the generation of models and validation of identified genes. For the present study, we developed and utilized a large subset of partially edited and augmented HMM profiles representing 37 C2H2 ZNF gene families within the bilaterian organisms and then used these profiles to predict gene family memberships from an extensive variety of 124 completely sequenced eukaryotic species.

## Results and discussion

The hybrid method developed for homolog detection is largely automated, rapid, and efficient for identifying members of C2H2 ZNF genes. This method utilizes HMM profiles of the gene families for initial sensitive detection of putative homologs from a variety of genomes and then validates these putative homologs using a focused BLAST search of a restricted set of well annotated genomes and comparison to a master list of known homologs. This method is logically extensible to any number of gene families represented by an HMM, and any number of complete genomes (and predicted proteomes) available for analysis. The NCBI refseq database and Swiss-prot provided an excellent resource for the C2H2 ZNF protein sequences used to generate HMM profiles after alignment of reference sequences. Since the entire analysis was dependent on the HMM profile, the quality of the profiles used is crucial. Care was taken to choose only families that have representative profiles. A total of 37 HMM profiles were generated for C2H2 ZNF gene families based on the existing information on those families.

During the first round of ortholog detection, 124 protein datasets belonging to various eukaryotic groups were scanned using 37 C2H2 ZNF HMM profiles separately. The output obtained consisted of potential homologs recognized for each profile within each genome. In the next step, a focused local BLAST used these potential profile-derived homologs individually as queries against a set of well annotated, reference genomes. The BLAST outputs generated were then scanned for the presence of “master list” genes as the top hits in order to decide unambiguous membership in the gene families represented by the HMMs used. The sequences found this way were used to further refine the HMM profiles to increase the specificity, and two more rounds of this process were performed. The final list of presumed gene family members was catalogued in a spreadsheet.

**Figure 1:**
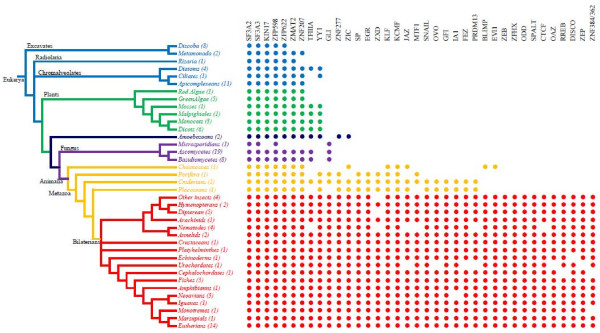
**Summarized representation of the distribution of 37 families among various groups of eukaryotes.** Numbers in the parentheses indicate the number of species under each phylum. Colors indicate various taxonomical groups (Light blue: Protists, Green: Plants, Dark blue: Amoebozoans, Purple: Fungus; Yellow: Lower metazoans and Red: Bilaterians). Family names are based on standard bilaterian family names as used in our previous publication [4].

The final output with 37 HMM profiles on 124 eukaryotic genomes identified 3,890 members of a relatively complex subset of C2H2 ZNF gene families. All identified C2H2 ZNF genes and their numbers across the tree of life are provided as spreadsheet in Additional files 1 and 2 respectively. The profiles generated in this study and the sequences identified are also provided as Additional file 3 and 4. Although initial HMM profiles were biased with more bilaterian sequences, subsequent scans employed separate HMM profiles for various eukaryotic groups derived from the sequences belonging to the respective groups. In the present study, 124 genomes were classified as 4 different groups. The first group included 30 species of protists belonging to excavates (including phyla Parabasilia, Fornicate and Euglenozoa), Chromalveolates (including phyla Apicomplexa, Ciliophora, Rhizaria, Heterokontophyta and Cryptophyta) and Amebozoa. The second group consisted of 16 different plant species belonging to Cyanidiophyta, Chlorophyta and Streptophyta. The third group had 28 species of fungi with phyla Basidiomycota, Ascomycota and Microsporidia. The last and the largest group consisted of metazoans with 50 species consisting of Choanozoa, Placozoa, Porifera, Cnidarian, Nematoda, Annelida, Arthropoda, Echinodermata, Tunicata, Cephalochordata and chordate (Additional file 5 and 6). Heterogeneous representation of various groups was mainly due to either a lack of genome sequence or non-availability of the proteome datasets. Despite the breadth of the organisms scanned, the results (Figure 1) indicate a clear pattern of gene block conservation within closely related organisms as well as a reasonable progression of gene family additions that correlates well with a presumed increase in organismal complexity. This nearly uniform block pattern was occasionally disrupted by the presence of “holes” within the blocks (perhaps representing a lineage or organism specific gene loss) and the presence of “loner” genes (genes that appear to be absent from almost all other closely related organisms). The latter may represent putative horizontal gene transfer events.

### Gene families present in all eukaryotes

Among the 37 C2H2 ZNF gene families, seven families (SF3A2, SF3A3, KIN17, ZFP598, ZFP622, ZMAT2 and ZNF207) appear to be present in almost all eukaryotes. Some exceptions include Discoba, which lacked families ZMAT2 and ZNF207, Rizaria, which lacked the ZNF207 family and microsporidia, which lacked ZMAT2, ZNF207, ZF622 and KIN17 families. A phylum/class represented by multiple species would be considered to have a particular family even if one organism belonging to the phylum lacked that gene family. Missing family members in some species could merely represent the absence of gene models from the data set due to error, incomplete sequencing coverage, or incorrect gene model prediction.

All 7 of these gene families have just one homolog in almost all the species scanned. SF3A3 (Splicosome factor 3a subunit 3), SF3A2 (Splicosome factor 3a subunit 2), Kin17, and ZMAT2 (Zinc finger Matrin Type 2) all encode single highly conserved U1-like C2H2 zinc finger domain. ZNF598 (Zinc Finger 598) has five C2H2 zinc finger domains, ZNF622 (Zinc finger 622) has four C2H2 zinc finger domains and ZNF207 has 2 C2H2 type zinc finger domains. SF3A3 and SF3A2 are known to act as subunits for RNA splicing machinery [16-18], Kin17 is believed to be involved in the cellular response to DNA damage, gene expression, and DNA replication, and ZNF622 is known to be involved in early T cell activation and embryonic development in mouse. The exact functions of the other gene families (ZNF207, ZNF598 and ZMAT2) are not clearly understood.

### The gene families added in plants and amoebozoans

The next expansion of gene families occurred in plants with an addition of the 2 families TFIIIA and YY1. Although lower plants belonging to phylum Chlorophyta (green algae) lacked these families, both families were present in all higher plants belonging to phylum Streptophyta. These families were represented as single homologs in most of the species, except YY1 which had 2 homologs in class Lillopsida. In addition to TFIIIA and YY1, Amoebozoa also had two more families, ZNF277 and ZIC. Though these families were not present in any other closely related groups (fungi or plants), they were present in lower metazoans.

TFIIIA (Transcription factor III A), with 9 zinc-finger domains, is a DNA-binding transcription factor known to bind RNA and required for 5sRNA gene expression in metazoans [4]. YY1 (Yin Yang 1) generally has 4 zinc-finger domains and has multiple functions, both as repressor and as an activator of gene expression [19]. In metazoans, they play roles in induction and patterning of the embryonic nervous system, differentiation within blood cell lineages, cell-cycle control, cell proliferation, differentiation, apoptosis, DNA synthesis and packaging, and X-inactivation [19]. The exact role of both these families in plants is not well understood.

### Gene family additions in fungi

Expansion of gene families in fungus included the addition of 2 families (TFIIIA and GLI) to the original 7 families present in all the eukaryotes. Of the 2 families, TFIIIA was also present in plants, while GLI was not. TFIIIA has just one homolog in all the fungus species, as is true for plants and other eukaryotes. Although GLI (Glioma-associated oncogene) occurs as a multi-gene family in most metazoans, it has one homolog in all fungus species. In Humans, the GLI family is known to regulate various aspects of early development of the central nervous system.

### Gene family additions in metazoans

The final massive expansion of C2H2 ZNF gene families occurred in metazoans with the addition of the remaining 25 gene families (Figure 1). Lower metazoans including Choanozoa, Porifera, Cnideria, and Placozoa only have a partial representation of these 25 families. Choanozoa, considered to be the most basal among the metazoans [20], have just 4 families added (KLF, JAZ, BLIMP and EVI1). They also lacked the families that were added in plants and fungi (GLI, TFIIA and YY1). The Porifera phylum, has an additional 4 families (SP, EGR, ZXD and MTF1), but compared to Choanozoa, they share just one family (KLF) and lack 3 families (JAZ, BLIMP and EVI1). Cniderians have all the families present in Choanozoa and Porifera except BLIMP and EVI1. They also have an additional 6 families (SNAIL, OVO, GFI, IA1, FEZ and PRDM13) not present in Choanozoa and Porifera. Placozoa have all the families present in cniderians except MTF1, GLI and TFIIIA. Most of the bilaterians have almost all the 25 families represented except for a few phyla/classes that lack one or more families. The prominent phyla/classes lacking the largest number of families are nematodes (lacking TFIIIA, ZXD, MTF1, JAZ, PRDM13, and CTCF) and urochordates (lacking JAZ, OVO, FEZ, PRDM13, ZEP and OAZ). Our observations on a large number of family losses in nematodes was consistent with a previous study [2,4]. Also, previously observed massive gene losses during the rapid evolution and adaptation of urochordates to a specialized environment could be the reason for the missing C2H2 ZNF gene families in urochordates [21-23]. Other phyla/classes lacking one or more families include arachnids (lacking ZNF 384), some insects (lacking ZXD), echinoderms (lacking DISCO), cephalochordates (lacking RREB, ZNF 384/362), neoavians (lacking IA1) and monotremes (lacking ZNF 384/362). The complete list of bilaterian specific zinc finger families are ZNF384/ZNP362, ZEP, DISCO (Disconnected), RREB (RAS responsive element binding protein), OAZ (Smad- and Olf-interacting zinc finger protein), CTCF, OSR (odd-skipped-related), SPALT, ZFHX1 and ZEB.

## Conclusions

Our approach combined features of both RBH and HMM methods of homolog detection. This technique is much faster than manual methods and is able to detect homologs accurately when compared to RBH alone [4]. Furthermore, this method can be easily applied to new gene families that can be represented by an HMM, and to any number of completed genomes (and predicted proteomes) available for analysis. A total of 3,890 genes was identified from 124 completely sequenced eukaryotic genomes that belong to 37 members of a relatively complex subset of C2H2 ZNF gene families. These gene families in eukaryotes revealed a stepwise evolutionary process of gene block additions, which agrees well with the phylogenetic relationship of the organisms [20], as well as a presumed increase in organismal complexity.

Out of the 37 total families, 7 families are present in all eukaryotes. The increased morphological complexity from primitive protists to plants or fungi involved addition of two families, with one family common to both fungus and plants. The final expansion in metazoans added 25 families to those present in other groups (protists, plants and fungi) and this expansion correlates with the large increase in morphological complexity of these organisms. Although choosing bilaterian conserved gene families to scan the other eukaryotes made this study biased towards bilaterians, it also allowed us to specifically trace the appearance, deletion, and expansion of these families during the course of eukaryotic evolution. Most gene families resistant to expansion (single member gene families) are highly conserved and are represented in most of the eukaryotic species. We assume that these families are present in the common ancestor of eukaryotes as they are involved in fundamental processes such as DNA damage repair and intron splicing. The remarkable conservation of these gene families with respect to sequence, as well as their ability to resist expansion, is consistent with previous observations [24-27]. Those functioning as structural proteins, pathogen response proteins, stress related proteins, signalling proteins, and proteins acting as transcription factors are often more prone to lineage specific expansions than are proteins that are involved in basic cellular functions like DNA modification and RNA metabolism [28]. It is still unclear why specific gene families undergo massive expansion while some remain unchanged across evolutionary distances. In general, C2H2 ZNF gene families with one or two ZNF domains are more resistant to expansion while multi ZNF domain containing families are not. It is assumed that such expansion occurs due to the modular structure of the multi-ZNF containing genes that provides a favourable platform for developing novel functionalities [29]. It has been hypothesized that lineage-specific expansions are a principle means of adaptation and one of the most important sources of organizational and regulatory diversity in many organisms during transitions towards higher complexity [28].

## Methods

### Generating HMM profiles

Previously identified putative orthologs that were present in the common ancestor of the bilaterians *Homo sapiens, Drosophila melanogaster*, and *Caenorhabditis elegans* were used as a focus for the present study [2,4]. For each of those families described, additional members belonging to various bilaterians that had well annotated genomes were collected from databases such as NCBI refseq [30] and Swissprot [31]. Both, similarity based searches as well as key-word based searches were used to retrieve the sequences. These families were further augmented with additional validated gene members described in the literature [3,4,32-34]. These sequences were aligned using the MUSCLE multiple sequence aligner program. The Hidden–Markov model (HMM) profiles for each of these families were created with the *hmmbuild* option of the HMMER 3.0 [35] package. The reference sequences were obtained from diverse taxa in order to make the profiles more representative of the genomes chosen for study.

### Obtaining eukaryotic protein datasets

The protein datasets of completely sequenced organisms representing all major eukaryotic clades were downloaded from NCBI, Ensembl, JGI, and Sanger. The downloaded genomes were then categorized into various class/phyla based on NCBI taxonomy information. Complete lists of the included species are given in Additional file 5, and the sources for these genomes along with their build numbers are provided in Additional file 6. The obtained genomes were sorted taxonomically into 4 groups as protists, plants, fungi and metazoans.

### HMM profile search

Whole predicted proteomes of the various species were scanned with all created HMM profiles using the *hmmsearch* option of the HMMER 3.0 package using minimum e-value threshold of 0.001. A loop written in Bash script was used to complete the reiterative *hmmsearch* procedure and the processing of results. For each HMM-genome pair, sequence hits were sorted based on the score for the full sequence and then on the best domain score. Only those sequences that had scores greater than 100 were chosen to be used in BLAST searches (standalone BLAST version 2.2.25. from NCBI) [7].

### BLAST search

Standalone BLAST was performed using the chosen sequences against a local sequence database consisting only of the well annotated, complete set of genes from *Homo sapiens, Drosophila melanogaster*, and *Caenorhabditis elegans.* Subsequently, no more than 3 best hits from these focused BLAST results were scanned for accession numbers that matched a master list of such numbers. This master list was constructed using only those genes from the three reference organisms that were members of a given HMM profile/family. This pairwise process was repeated for each profile and each genome. Only the sequences that identified the correct family as verified by the master list accession numbers were chosen as family members.

### Increasing specificity

The process was repeated two more times after adding the identified members from the previous round to generate a new HMM profile for the family. In order to increase the specificity of ortholog detection, during the second round, separate HMM profiles were generated for each of four taxonomic clades protists, fungi, plants, and metazoans. For those families for which the sequence data was not available for different clades, general profiles were again used in the second round after updating the HMM profile with new sequences.

We carefully re-examined all the species lacking families that were otherwise present in closely related species of the group/clade. To make sure that these families were likely to be missing rather than just difficult to identify not because of the poor quality of the proteome or poor annotation, we performed a focused blastp/tblastn search of these proteomes/genomes using sequences from closely related species. If no members were found after two rounds of HMM profile search and focussed BLAST search, the family was declared missing from the species. All the sequences identified as orthologs in the respective family were then catalogued. Those families that had multiple members were then analysed to determine whether they were truly parologous or just duplicate sequences by aligning them using clustalw software [36].

## Competing interests

The authors declare that they have no competing interests.

## Authors' contributions

GS established the overall concept and approach, and AS initiated gene identification, organization, and documentation of genes, as well as producing all tables and writing early drafts of the manuscript. All authors read and approved the final manuscript.

## Supplementary Material

Additional file 1 **Sequence identifiers for the genes identified as C2H2 ZNF family members.** The database for these gene IDs is provided in Additional file 6. Click here for file

Additional file 2 **Number of C2H2 zinc finger genes in each of the 37 gene families identified from 124 different organisms.** The genomes are listed in the phylogenetic order and block wise appearance of the genes are depicted as colored cells. Colors indicate various taxonomical groups (Light blue: Protists, Green: Plants, Dark blue: Amoebozoans, Purple: Fungus; Yellow: Lower metazoans and Red: Bilaterians). Click here for file

Additional file 3 ZIP file containing all the HMM profiles used in the study. Click here for file

Additional file 4 **ZIP file containing protein sequences identified as C2H2 zinc finger family members.** They are named as genus_spp_ family_ geneID.fasta. Click here for file

Additional file 5 List of species represented in different phyla/class of the protists, plants fungus and metazoans. Click here for file

Additional file 6 List of species used in the current study along with their genome build and source. Click here for file
